# Who uses firearms as a means of suicide? A population study exploring firearm accessibility and method choice

**DOI:** 10.1186/1741-7015-7-52

**Published:** 2009-09-24

**Authors:** Helen Klieve, Jerneja Sveticic, Diego De Leo

**Affiliations:** 1 Australian Institute for Suicide Research and Prevention, National Centre of Excellence in Suicide Prevention, WHO Collaborating Centre for Research and Training in Suicide Prevention, Griffith University, Brisbane, Australia

## Abstract

**Background:**

The 1996 Australian National Firearms Agreement introduced strict access limitations. However, reports on the effectiveness of the new legislation are conflicting. This study, accessing all cases of suicide 1997-2004, explores factors which may impact on the choice of firearms as a suicide method, including current licence possession and previous history of legal access.

**Methods:**

Detailed information on all Queensland suicides (1997-2004) was obtained from the Queensland Suicide Register, with additional details of firearm licence history accessed from the Firearm Registry (Queensland Police Service). Cases were compared against licence history and method choice (firearms or other method). Odds ratios (OR) assessed the risk of firearms suicide and suicide by any method against licence history. A logistic regression was undertaken identifying factors significant in those most likely to use firearms in suicide.

**Results:**

The rate of suicide using firearms in those with a current license (10.92 per 100,000) far exceeded the rate in those with no license history (1.03 per 100,000). Those with a license history had a far higher rate of suicide (30.41 per 100,000) compared to that of all suicides (15.39 per 100,000). Additionally, a history of firearms licence (current or present) was found to more than double the risk of suicide by any means (OR = 2.09, *P *< 0.001). The group with the highest risk of selecting firearms to suicide were older males from rural locations.

**Conclusion:**

Accessibility and familiarity with firearms represent critical elements in determining the choice of method. Further licensing restrictions and the implementation of more stringent secure storage requirements are likely to reduce the overall familiarity with firearms in the community and contribute to reductions in rates of suicide.

## Background

Significant declines in the use of firearms as a means of suicide are widely documented [1] with identified links to the introduction of access restrictions [2-6]. However, conflicting reports on the effectiveness of such restrictions [7,8] support the call for a broader explanation for access to and the use of firearms in suicide.

### Accessibility

Assessments of firearm access have adopted different approaches: while, in some cases, availability has been measured directly by household surveys [9,10] or the number of gun licences [3], investigations have more often used proxy measures such as the number of purchased handguns [11], gun magazines subscriptions [12,13] or comparison of rates of firearms suicides [8]. Disparities between studies also exist with regard to the definition of 'access'. For example, Ikeda *et al*., using data from the USA Injury Control and Risk Survey, examined details of 5056 respondents who provided information on their firearms ownership and access. They reported that 313 (6.2%) respondents owned firearms but did not identify the possibility of rapid access to them, while 421 (8.3%) did not personally own firearms but could have rapid access to them, with women and members of ethnic minorities having the greatest representation in the latter group [14]. Similarly, Smith in the 2001 USA National Gun Policy Survey found that while only 24.1% of respondents personally owned a gun, 34.9% had a firearm within the household, with women again showing the greatest level of accessibility without ownership (at an almost three times the difference) [15].

Unlicensed access is particularly difficult to identify - this including firearms licensed to others, for example family members, and illegal and unlicensed weapons. One method by which these can be obtained is through the theft or purchase of stolen firearms. A 2005 to 2006 study on firearm theft in Australia reported 302 stolen firearms, mainly rifles in Queensland, with 7% of these being unregistered. Storage was identified as a major issue, with 22% of 'firearm only' thefts were from unsecured premises with a further 27% obtained by 'entry undetermined' [16].

### Suicide risk

While recognizing varying data quality, the majority of studies confirm the positive association between firearm access and increased firearm suicide risk [3,17,18], with the strongest relationship for those having firearms at home [1,19-23]. However, studies on the association between firearm access and the total suicide rates have not been completely conclusive. In fact, a number of investigations have found significant correlations between firearms accessibility and increased suicide rates [18,19,21,22,24,25], while others could not confirm that firearms availability in itself increases suicide rates [13,17,26].

In general, higher rates of firearm suicide were found in geographical areas with greater firearms ownership and less restrictive firearms law, most notably in the USA [22,27]. Rural and regional differences (i.e. areas outside cities) were also identified in Australia, with greater regional declines in rates of firearm suicide identified following the imposition of access restrictions, even if the rates continued to remain higher in the same areas [28].

### Method choice

There have been widely reported successes in strategies restricting access to means of suicide, for example, after the detoxification of domestic gas [29] or placement of barriers on frequent sites of suicide by jumping [30]. In these cases, no substitutions were detected and the overall suicide rates were positively influenced, at least initially [17]. It has been suggested that means substitution has accounted for the decrease in suicide using firearms but increased the rate of suicide by hanging in Australia. However, a closer analysis of the data failed to confirm the substitution theory [31], but rather emphasised the need to consider more complex factors behind changes in the choice of method (e.g. social acceptability, change in attitudes, etc.).

The effect of 'instrumentality', which suggests that those with a strong propensity for one method would not seek a substitute [4,12], should also be noted. Links between demographic and psychological characteristics [32-34] and socio-cultural acceptability of certain suicide methods [35] are increasingly recognized (see the choice structuring model by Clarke and Lester [36]).

Using detailed data for each suicide case matched to its history of legal firearm possession adds to the current debate on the effectiveness of firearm restrictions in Australia [6,7,37]. It provides a unique method of examining the patterns underlying the choice of suicide methods for those with or without legal access to firearms and the associated suicide risk in these groups.

## Methods

Data on suicides in Queensland were accessed from the Queensland Suicide Register (QSR), a comprehensive database of suicide mortality data from 1990 to present day, which contains information derived from police reports on all suicides in Queensland (including autopsy and toxicology reports) [38]. The use of data from the QSR has continuing ethnical approval from the Griffith University Ethics Committee.

For this study, the analysis included all 'probable' or 'beyond reasonable doubt' cases of suicide during the years 1997-2004 (*n *= 4,469). Queensland population data, 2001, obtained from the Australian Institute of Health and Welfare [39] were used for the calculation of the rates. Table 1 provides details of licence possession and suicide incidences in Queensland.

**Table 1:** Number of firearm licences and the incidence of suicide in Queensland, 2001.

Population	3,628,946
Number of suicides	1 91,109

By any method	505
By firearms	60
By hanging	234

(Sources: Queensland Suicide Register, State and Territories GRIM (general record of the incidence of mortality) Books, Queensland Firearms Registry.)

In Australia, the possession of a firearm licence requires that applicants provide details of how the firearm will be used, including its use in farming activities. Therefore, it can be assumed that a current licence implies both familiarity and accessibility as a result of the need for specified use and regular renewals of the licence. A data matching process, focused on the name, date of birth, age and address of the licence holder, was undertaken between the QSR and the Firearms Registry (Queensland Police Service), which identified the licence history of all suicide cases from 1997 onwards. Where partial matches occurred (for example, an unspecified birth date, a different address between the time of death and the application for the licence), an additional review of original the QSR file was undertaken.

Geographical location has been found to influence use of firearms in suicides [28]. The Accessibility/Remoteness Index of Australia (ARIA Index) provides a measure of the accessibility of regional localities [40]. An ARIA score was identified for each case, with scores between 0 and 3.51 defining Highly Accessible/Accessible localities such as major cities, specified as ARIA1, and scores greater than 3.51 defining Moderately Accessible, Remote or Very Remote localities, classed as ARIA2. Blood alcohol levels at death were accessed from toxicology reports.

### Statistical analyses

This study describes all known cases of suicide in Queensland with these matched against known licence possessions - thus describing the population of suicide cases in Queensland 1997-2004.

Descriptive analysis was used to compare the characteristics of those with a history of licence possession and the use of firearms in suicide firearms/other. The comparison of these groups provides a link between firearm accessibility and the chosen method. Rates of suicide were then used to document population differences by method used and availability of means, based on known licence history.

Odds ratios (OR) were used to assess the suicide probability, with confidence intervals and significance tests evaluated using Woolf's equation [41]. This analysis was conducted between familiarity with firearms by licence history and outcome (suicide or not). The proportion of the population who were familiar with firearms was estimated setting the number of people in Queensland who had a licence in 2001 (*n *= 191,109, accessed through the Queensland Firearms Registry) against the remainder of the 2001 general population (*n *= 3,437,837). Finally, a logistic regression, with independent variables entered simultaneously, was conducted using predictors of holding a current licence, gender, age group, ARIA group, positive blood alcohol level and marital status. All statistical analyses were undertaken using SPSS Version 15. The two-tailed significance was set at 5%.

## Results

Table 2 details the demographic features of suicide cases based on licence history and method choice (firearms or other). The characteristics of those with a current licence were, in most cases, very similar to those who used firearms as a method of suicide. Far more men had a licence (95.3%) than women, and men used firearms as a means of committing suicide more often than any other method (93.3%). There was also an obvious age difference in both licence possession and the method of suicide, as younger people were less likely to have a licence (2.4% to 11.2% for under 25 years and 65+ years, respectively) and less likely to use firearms as a means of committing suicide (7.6% to 23.5% for under 25 years and 65+ years, respectively).

**Table 2:** Demographic factors of all suicide cases presented by licence history and suicide method.

		**Licence history**			**Suicide method**	
	
		**At death**	**In the past**	**No record**	**Firearms**	**Other**
Gender	Male	262 (95.3%)	183 (96.8%)	3100 (77.4%)	461 (93.3%)	3084 (77.6%)
	
	Female	13 (4.7%)	6 (3.2%)	905 (22.6%)	33 (6.7%)	891 (22.4%)

Age	<25	18 (2.4%)	3 (0.4%)	742 (97.2%)	58 (7.6%)	705 (92.4%)
	
	25-64	200 (6.3%)	143 (4.5%)	2,849 (89.2%)	317 (9.9%)	2,875 (90.1%)
	
	65+	57 (11.2%)	43 (8.5%)	407 (80.3%)	119 (23.5%)	388 (76.5%)

	Married/*De facto*	134 (54.7%)	74 (46.2%)	1,246 (37.9%)	201 (47.4%)	1,253 (38.4%)

	Blood alcohol level = 0.05 ppm	163 (23.5%)	126 (20.3%)	2,386 (29.1%)	278 (24.3%)	2,397 (28.8%)

	ARIA2	192 (29.9%)	147 (21.8%)	3,232 (17.2%)	337 (30.8%)	3,234 (16.6%)

(Note: Comparisons are between all groups with those with a licence at time of death.)

ARIA2 = areas listed as moderately accessible, remote or very remote areas in the

Accessibility/Remoteness Index of Australia

Higher proportions of those with a current licence were married (54.7%, compared to 37.9% with no licence history) and lived in rural areas (29.1% classed as ARIA2, compared to 17.2% with no licence history). These patterns were similar when comparing those using firearms as a means of suicide to those using other methods - 47.4% were married (compared to 38.4%) and 30.8% lived in rural areas (compared to 16.6%). Finally, those without a licence history were slightly more likely to have a blood alcohol level of above 0.05 ppm than current licensees (29.1% and 23.5%, respectively), as were those who chose other suicide method compared to firearms suicides (28.8% and 24.3%, respectively). Those who had held a licence in the past were likely to be slightly older and less likely to be rurally located - only 21.8% were recorded as living in ARIA2, compared to 29.9% of current licensees.

**Figure 1:**
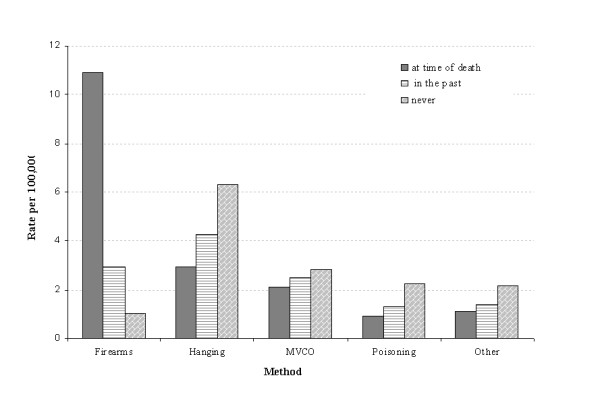
**Method selection rates in persons by firearms licence history**.

Marked variations in the methods selected were observed in those who had a firearm licence history (Figure 1). Two hundred and eight-two people (1.03 per 100,000) who did not have a licence history used firearms as a means of suicide, which is a far lower rate than for those with a current (10.92 per 1000, 000) or past licence history (2.94 per 100,000). The rate of all suicides by those with a licence history was 30.41 per 100,000, almost double the rate of 15.39 per 100,000 for all suicides.

The relationship between firearm familiarity (those with a licence history) and the likelihood of their committing suicide is explored in Table 3. This shows a very highly significant relationship between those who are familiar with firearms and the risk of suicide (OR = 2.09, *P *< 0.001), expressing double suicide rate in those with an identified exposure to weapons.

**Table 3:** Risk of suicide on the basis of licence possession.

		**Suicide Risk**					
					
		**Suicide**	**Control**	**z**	**Odds ratio**	**95% confidence interval**	
History of firearm licence	Yes	464	190645	14.98	2.09	1.90	2.30
					
	No	4005	3433832				

A logistic regression analysis (Table 4) was undertaken in order to study the factors found to be associated with using firearms as a means of suicide. The strongest relationship to the choice of firearms as a method was a current licence (OR = 14.16, *P *< 0.001). Age and gender (65+ years: OR = 2.81, *P *< 0.001; males: OR = 2.52, *p *< 0.001) were also significant factors. Whilst being married or having a positive blood alcohol level were not significant, accessibility indicated a greater likelihood of those in more regional and remote areas to use firearms (OR = 2.26, *P *< 0.05).

**Table 4:** Logistic regression results showing predictors of the selection of firearms as a suicide method.

		**Confidence Interval**	
**Variables in the equation**	**Exp b #**	**Lower**	**Upper**
Age:			
under 25†			
25-64	0.945	.630	1.418
65+	2.805***	1.737	4.532
Gender			
Male/Female†	2.519***	1.606	3.952
Licence at death:			
Yes/Never†	14.160***	10.040	19.970
Accessibility:			
ARIA1†/ARIA2	2.261*	1.641	3.113
Married:			
Yes/Other†	1.216	0.917	1.615
Blood alcohol ≥ 0.05:			
Yes/No†	0.899	0.656	1.232
Final model:			
Cox and Snell R^2 ^= 0.116, Nagalkerke R^2 ^= 0.245, *χ*^2^(1) = 354.023, *p *< 0.001			

# * *p *< 0.05, ** *p *< 0.01, *** *p *< 0.001

† Reference category

The main deviations from the identified relationship between those with a current licence and the selection of firearms as a suicide method occurred: (i) where an available firearm was not used (*n *= 108); or (ii) where those without a licence history used a weapon to commit suicide (*n *= 282). Table 5 summarizes the characteristics of these groups.

**Table 5:** Incidence of suicide where availability or use are not consistent with licence status.

		**Age**			
		
		**<25**	**25-64**	**65+**	**Comparison ARIA1/ARIA2**
Current firearm licence - other suicide method used					

Males	ARIA1	9 (60.0%) *n *= 15	61 (45.2%) *n *= 135	9 (26.5%) *n *= 34	79 (42.9%) *n *= 184
	
	ARIA2	0 (0%) *n *= 3	17 (30.9%) *n *= 55	5 (26.3%) *n *= 19	22 (28.6%) *n *= 77

					

Females	ARIA1	0 (0%) *n *= 0	4 (50.0%) *n *= 8	0 (0%) *n *= 0	4 (50.0%) *n *= 8
	
	ARIA2	0 (0%) *n *= 0	1 (50.0%) *n *= 2	2 (66.7%) *n *= 3	3 (60.0%) *n *= 5

No licence history - firearm suicide method used					

Males	ARIA1	25 (5.8%) *n *= 433	116 (6.5%) *n *= 1,776	43 (16.5%) *n *= 261	184 (7.4%) *n *= 2470
	
	ARIA2	15 (11.4%) *n *= 132	37 (10.9%) *n *= 341	15 (25.9%) *n *= 58	67 (12.6%) *n *= 531

					

Females	ARIA1	5 (4.1%) *n *= 121	8 (1.4%) *n *= 558	3 (3.9%) *n *= 77	16 (2.1%) *n *= 756
	
	ARIA2	3 (7.9%) *n *= 38	6 (6.4%) *n *= 94	0 (0%) *n *= 7	9 (6.5%) *n *= 139

ARIA = Accessibility/Remoteness Index of Australia; ARIA1 = highly accessible or accessible localities; ARIA2 = moderately accessible, remote or very remote localities.

(Note: data presented by observed number in group, % of group and total number in group (all methods). Six cases without a licence could not have their ARIA score determined.)

Among males with a licence, a greater proportion of those younger than 25 years of age used an alternative method - 60.0%, compared to 26.5% of those aged 65+ years. Also a greater proportion of ARIA1 men selected alternative means (42.9%, compared to 28.6% for ARIA2). For those without a licence history, but who had used firearms as a means of committing suicide, a greater proportion of both men and women were from ARIA2 (12.6% and 6.5%, respectively). In general, women are far less likely to use firearms as a suicide method which makes it more difficult to identify any significant trends. However, it is interesting to note that of the 13 females with a licence, seven used an alternative method. Twenty-five females with no identifiable licence were recorded as using firearms to suicide, suggesting they may have had access to firearms licensed to another person in their home [15].

## Discussion

This research, drawing on the evidence of firearms suicides during 1997 to 2004, furthers the understanding of the relationship between access to means, method familiarity and method choice. It also supports more recent work arguing for a broader explanation of factors (including socidemographic) impacting on the use of firearms as a means of suicide [7]. It demonstrates that not only is there a greater likelihood that those familiar with firearms and having access to them would select firearms as a means for suicide, but also that there is a greater likelihood of suicide by any means among this group.

Results from the logistic regression identified significant factors for those most commonly selecting firearms as a method of suicide. These included age, location and firearm access, with older males from regional areas with access to a firearm being the most likely group to use firearms to commit suicide.

The presence of firearms has been found to be associated with a significantly higher risk of suicide by the use of firearms in men than women. This is consistent with some other studies [42]. Additionally, this research found that the risk of suicide by any means was more than doubled in those with a licence history (OR = 2.09, *P *< 0.001).

A number of other studies have explored the link between suicide and firearm access. At a regional level, associations between higher firearm availability and total suicide rates have been identified [21,22,25]. However, small samples and the effects of overall firearm accessibility often make it difficult to confirm the relationship. Beautrais *et al*. [17], in a study of 197 suicide cases in New Zealand, found a positive, but non-significant, association between firearm access in the home and the selection of this means of suicide (OR = 1.4, *P *> 0.05). Kellerman *et al*. [20] found an increased suicide risk (OR = 4.8) where a firearm was available in the home. Studies focusing on links between recent firearm access and suicide risk also found an increased risk among those who had recently purchased handguns [11,43].

Given the significance of the finding of an elevated suicide risk in those with a history of firearm access, some consideration of the possible differences in this group is warranted. Few studies have explored the mental health [44] and rates of depression and suicide ideation [45] among those with access to firearms, but found no significant links. Gun owners are known to be a relatively consistent demographic group [46]. In our study the profile of those choosing firearms as a means of suicide showed them to be older regional men, many of whom were farmers. The higher incidence of suicides in older men, and in regional localities, is well documented [38], as are the higher rates among Australian farmers [47,48]. In seeking to explain the higher suicide rates of farmers in the UK, Booth *et al*. suggested a number of factors, including a more functional attitude to death associated with the nature of the farming lifestyle [49]. Such attitudes are likely to be reinforced over time, with Hendrix [46] suggesting a cultural transmission of values surrounding firearms. There is a need for further exploration of these relationships with elevated suicide rates.

### Method choice: access versus selection

The present study (Figure 1) showed that, amongst current licensees, firearms remained the preferred method of suicide (10.92 per 100,000), in contrast to those without a licence history for whom hanging was the most preferred method (6.31 per 100,000) and firearms the least preferred (1.03 per 100,000). Further, for those with documented familiarity with firearms, holding a current or past licence, there was a greater likelihood of their committing suicide compared to those without such a history (OR = 2.09, P < 0.001).

Of particular interest were individuals with an identified access to firearms who used other means of suicide (*n *= 108). They were more likely to be younger people from ARIA1 areas - thus poorly aligning with the more usual characteristic of firearm suicides. Such a choice (by seven of the 15 males aged less than 25 years from ARIA1) is consistent with the increasing choice of hanging among young males, both in Australia [31] and internationally [50].

The evidence shows that those who accessed firearms without a licence were significantly more likely to be older, male and from ARIA2. This group aligns well with the profile of those selecting firearms, with the exception of current licence holders. There was no significant age difference for females; however, they were significantly more often from ARIA2 (Table 5).

While the access restrictions introduced in 1996 were intended to limit firearm use to those with licences, there are a number of likely avenues by which illegal access can be achieved - for example, through access to firearms in the home, through work or social networks or through theft. Studies by Smith [15] and Ikeda *et al*. [14] documented access without ownership, particularly by females and young males, who were the least likely to hold a licence. In their investigation of firearm theft, Bricknell and Mozous [16] showed that a greater proportion of Queensland thefts occurred in rural and regional areas, and that only 57% of licensed owners complied with the safe storage requirements. Thus it can be seen that it is relatively easy for unlicensed people to obtain access to firearms.

While accessibility is a critical element of choice, this research demonstrates that a licence is not a necessary factor in determining firearm accessibility. Further, where access is present through a licence, this may also not be a sufficient factor in determining the choice of the method of suicide. The relevant number of those using other means demonstrates that the choice of method is also greatly influenced by age and location, with illegal access occurring more frequently in regional locations.

### Limitations

Owning a current firearm licence has been used as a proxy for current access. Although this is a good indicator of access, there are situations where a person with a licence may not have immediate access, for example, when away from their normal residence. On the other hand, for those without a licence there are a number of mechanisms by which firearms can be accessed. Those with a past licence may still retain their old weapon. Further, those without a license can obtain weapons stored by known licence holders (sometimes within their household) or illegal weapons.

The identification of licensed persons used the most detailed information available, integrating information from the QSR with that from the Firearms Registry. However, it is possible that inaccurate reporting or recording of information could have occurred in the documentation of some cases.

Firearm suicide is predominantly a method chosen by males; thus, the numbers of reported female cases were low and the possibility of identifying statistical differences, for example across age classes, was limited.

This study relied on data from the QSR, limiting the analysis to Queensland. Similar analyses for all of Australia, while certainly possible, are constrained in their effectiveness by recognised issues with national data quality [51,52].

## Conclusion

This research suggests that familiarity with and access to firearms is central not only to the choice of this method, but is also linked to a greater likelihood of committing suicide by any method. Factors such as gender, age and geographical location impact on the choice of this means, with older regional males the most likely to choose firearms. It is apparent that access to firearms has been possible for a large number of persons without a current licence; however, a valid licence increases the likelihood of this choice as a suicide method, even if it is not an essential prerequisite.

In progressing firearms policy, the implementation of more stringent secure storage requirements and further licensing restrictions are likely to contribute to continued reductions in firearm suicides, and also reduce the overall familiarity with firearms in the community. While this study was limited to Queensland, its results are possibly relevant for other jurisdictions, particularly states with high prevalence of firearms suicides.

## Abbreviations

OR: odds ratio; QSR: Queensland Suicide Register; ARIA: Accessibility/Remoteness Index of Australia; ARIA1: highly accessible or accessible localities; ARIA2: moderately accessible, remote or very remote localities.

## Competing interests

The authors declare that they have no competing interests.

## Authors' contributions

HK participated in the design of the study, linking data from the QSR and the Firearm Registry, performed the statistical analysis and contributed to the writing of the paper. JS contributed to the data analysis and the writing of the paper. DDL conceived the study, participated in its design and helped to finalize the manuscript for publication. All authors read and approved the final manuscript.

## Pre-publication history

The pre-publication history for this paper can be accessed here:


